# Metabolomics in the identification of biomarkers of dietary intake

**DOI:** 10.5936/csbj.201301004

**Published:** 2013-02-07

**Authors:** Aoife O'Gorman, Helena Gibbons, Lorraine Brennan

**Affiliations:** a UCD Conway Institute, Belfield, Dublin, Ireland; b UCD Institute of Food and Health, Belfield, Dublin, Ireland

**Keywords:** Biomarkers, habitual diet, metabolomics

## Abstract

Traditional methods for assessing dietary exposure can be unreliable, with under reporting one of the main problems. In an attempt to overcome such problems there is increasing interest in identifying biomarkers of dietary intake to provide a more accurate measurement. Metabolomics is an analytical technique that aims to identify and quantify small metabolites. Recently, there has been an increased interest in the application of metabolomics coupled with statistical analysis for the identification of dietary biomarkers, with a number of putative biomarkers identified. This minireview focuses on metabolomics based approaches and highlights some of the key successes.

## Introduction

In today's modern world nutrition research is focused on improving population and individual health through diet [1]. Nutrition and health related research are beginning to understand that in addition to their essential functions, nutrients and non-nutrient components of foods interact with numerous metabolic pathways and influence health reducing or increasing the risk of disease. Diet is considered one of the major factors contributing to the rapid increase in the incidence of metabolic disorders such as obesity, diabetes and cardiovascular disease [2].

Reliable dietary assessment methods are vital when attempting to understand the links between diet and chronic disease profiles. Conventional tools for collecting quantitative information on dietary exposure such as food diaries, 24-h recalls and food frequency questionnaires (FFQ) can be unreliable for characterising and quantifying eating behaviour and are all subject to possible reporting and other biases [3, 4]. In addition, these methods are unreliable for certain groups such as the obese or elderly people, whose self-reported energy intakes tend to be underestimated, as assessed by energy expenditure measurements using the doubly labelled water method [5, 6]. A full critical review of limitations associated with the current techniques is beyond the scope of the present review and the reader is referred to the following papers [3, 4, 7–9].

In an attempt to overcome the problems with measuring dietary exposure with self-reported methods, nutritional epidemiologists started examining biomarkers as measures of dietary intake and nutrient status [10, 11]. The use of dietary biomarkers provides a more objective and accurate measure of intake in comparison to traditional questionnaires as they take into account the nutrient bioavailability and metabolism [12, 13]. One of the main applications of these dietary biomarkers is to use them as reference measurements to assess the validity of dietary assessment measures [11, 14, 15]. So far ideal biomarkers exist for salt and protein intake (sodium/nitrogen measure in a 24 h urine sample) and energy expenditure (double labelled water technique) [11]. Other biomarkers exist that do not provide information on the exact dietary intake but which are highly correlated with intake for example the measurement of serum cartenoids and vitamin C as biomarkers of fruit and vegetable intake [16].

The development of robust food biomarkers will help in better classifying a person's dietary intake and in turn will improve the assessment of the relationship between diet and chronic disease [17]. In recent years there has been an increased interest in applying metabolomics for the discovery of biomarkers of dietary intake. This review will focus on metabolomics and its use in assessing dietary biomarkers.

## Metabolomic Technologies

Metabolomics refers to comprehensive and non-selective analytical chemistry approaches aiming to provide a global description of all metabolites present in a biofluid at a given time [18–21]. The two main approaches employed in metabolomics are nuclear magnetic resonance (NMR) spectroscopy and mass spectroscopy (MS). These techniques both have their advantages and disadvantages and at present there is no single analytical technique capable of measuring and identifying all metabolites in a single sample simultaneously and therefore comprehensive metabolomic data needs to be assessed by bringing together data from different platforms [22].

For instance, NMR spectroscopy uses an untargeted approach where all molecules are interrogated simultaneously by properties that they all share (NMR active hydrogen or carbon) [23]. ^1^H NMR requires little or no pre-treatment, it is quantitative (absolute), non-destructive, reproducible and unbiased [24], although is not as sensitive as MS techniques. There are a range of MS based approaches, with the most recent advances leading to the use of hyphenated techniques such as liquid chromatography-mass spectroscopy (LC-MS), capillary electrophoresis-mass spectroscopy (CE-MS) and gas chromatography-mass spectroscopy (GC-MS). The chromatographic step allows the separation of metabolites before detection takes place. One of the main advantages of these techniques is the associated high sensitivity and therefore may detect metabolites that are present in a concentration below the detection limit of ^1^H NMR spectroscopy. A disadvantage is the necessity for sample preparation before analysis. GC-MS also requires derivatization, which lengthens the sample preparation time. As stated above, a comprehensive overview of the metabolome is achieved by using the multiple platforms.

## Data Analysis

Metabolomics like other ‘omic’ technologies generates large and complex datasets and because of this data analysis using multivariate statistics has become an important part of metabolomics. There are a number of statistical methods available for metabolomic data, with principal component analysis (PCA), partial least squares discriminant analysis (PLS-DA) and orthogonal PLS-DA being the most commonly used. PCA is probably the best known method, it is an unsupervised method which assesses the natural grouping of sample classes and can be used to identify extreme outliers [25].

Despite its widespread use in metabolomics, PCA has a number of shortcomings. Mainly, PCA does not have an associated probabilistic model, which makes assessing the fit of PCA to the data difficult, limiting the scope of its application. In addition PCA can fail to reveal underlying groups of subjects in the data, thereby providing a false view of the underlying data structure [26, 27]. Probabilistic principal component and covariates analysis (PPCCA) is a novel extension of probabilistic principal component analysis (PPCA) [28] which has recently been introduced to analyse metabolomic data. PPCCA incorporates covariates into the model and facilitates joint modelling of metabolomic data and covariates, meaning that the PPCA model directly models any variation due to the covariates, thus ensuring that the principal components provide a clear picture of the underlying data. This method has great potential for use within the metabolomics field [29].

Supervised techniques require prior knowledge of the class of a sample and examples frequently employed in metabolomics studies include, PLS-DA or O-PLS-DA, combining a data filtering step. PLS-DA provides a way to filter out metabolic information which is not correlated to the predefined classes and the loadings plots provide information on the spectral signals associated with the observed trends giving a means to interpret the metabolic information. Despite its powerful ability to separate classes, care must be taken during fitting of PLS-DA to the training detaining datasets, which exaggerate generalisation ability. Generally, cross-validation or permutation tests are required to assess the ability of the trained PLS-DA model [30]. For further information on these techniques and other analysis tools such as random forests (RF) [31], support vector machines (SVM) [32] and artificial neural networks (ANN) [33], please see the following recent reviews [34, 35].

## Metabolomic & Dietary Biomarker Studies

Applications of metabolomics to identify novel dietary biomarkers have in general terms taken three approaches (i) specific acute intervention to identify food markers (ii) searching for biomarkers in cohort studies and (iii) analysis of dietary patterns in conjunction with metabolomic profiles to identify nutritypes and biomarkers. Approaches (i) and (ii) form the basis of the studies described under biomarkers of specific foods while approach (iii) is discussed under dietary patterns.

### (i) Biomarkers of specific foods

Over the past few years a number of studies have emerged where specific acute interventions have been used to identify the presence of food specific biomarkers or to monitor concentration changes in diet related metabolites. To date, application of metabolomics has identified a number of putative biomarkers of intake of certain foods including salmon, broccoli, wholegrain wheat cereal, raspberry [36], cruciferous vegetables [37], citrus fruits [17], coffee [38, 39], onions [40] and red meat [41]. An initial literature search identified a number of biomarkers associated with a broad range of foods (meat, fish, wholegrains, cocoa etc.). However, for the purpose of this minireview we focused on the following foods for table 1: Meat, Fish, Vegetables, Citrus Fruits, Coffee and Tea.

**Table 1:** Examples of dietary biomarkers identified using metabolomic based approaches

Food	Sample	Metabolic Approach	Biomarker	Study
**Red Meat**	Urine	Ion exchange chromatography	1- and 3-methylhistidine	[42]
	Urine	^1^H NMR spectroscopy	O-acetylcarnitine, N,N-dimethylglycine	[43]
	Urine	^1^H NMR spectroscopy	O-acetylcarnitine	[44]
	Serum and urine	^1^H NMR spectroscopy	Creatine, histidine, urea	[45]
	Urine	^1^H NMR spectroscopy	Carnitine, Creatinine, TMAO, acetyl-carnitine, taurine, 1- and 3-methylhistidine	[41]
	Plasma	HPLC	Carnosine	[46]
	Urine	Ion exchange chromatography	Creatinine, taurine, 1- and 3-methylhistidine	[47]
**Cooked Meats**	Hair	LC–MS	PhIP	[48]
	Urine	LC-MS-MS	PhIP metabolites	[49]
	Urine	GC–MS	PhIP	[50]
	Urine	NCI-GC-MS^a^	PhIP	[51]
	Urine	GC-MS	4'-OH–PhIP	[52]
**Fish**	Urine	FIE-MS	TMAO, anserine , 1- and 3- methylhistidine	[36]
	Plasma	LC-MS-MS	Proline-hydroxyproline	[53]
	Urine	^1^H NMR spectroscopy	TMAO	[54-57]
**Vegetables**				
Cruciferous Vegetables	Urine	^1^H NMR spectroscopy	SMCSO	[37]
Vegetarian diet	Urine	^1^H NMR spectroscopy	Phenylacetylglutamine and glycine	[43]
	Urine	^1^H NMR spectroscopy	p-hydroxphenylacetate	[41]
	Urine	^1^H NMR spectroscopy	Hippurate, N-acetyl glycoprotein and succinate	[44]
	Urine	HPLC-ESI-MS-MS	Enterolactone and kaempferol	[58]
**Citrus Fruits**	Urine	^1^H NMR spectroscopy	Proline betaine	[17]
	Urine	FIE-MS	proline betaine and conjugates-sulphate	[59]
	Urine	HPLC-ESI-MS-MS	Naringenin, hesperetin and sulphonated derivatives of caffeic acid	[58]
**Coffee**	Urine	HPLC-ESI-MS-MS	Chlorogenic acid	[58]
	Serum	ESI-MS-MS	Sphingomyelins	[38]
	Plasma & urine	HPLC	Urinary dihydrocaffeic acid-3-0-sulphate & feroloyglycine	[39]
	Plasma	LC–MS–MS	3,4-Dimethoxycinnamic acid, 3,4-Dimethoxy dihydrocaffeic acid	[60]
**Black/Green Tea**	Urine	HPLC-MS-MS	Hippuric acid	[61]
	Urine	HPLC-FTMS^b^ and HPLC-TOFMS-SPE-NMR^c^	Hippuric acid and a structurally related hydroxybenzoic glycine conjugate, vanilloylglycine, and pyrogallol-2-O-sulfate	[62]
	Urine	^1^H NMR spectroscopy	Hippuric acid and 1,3-dihydroxyphenyl-2-O-sulfate	[63]
	Urine	^1^H NMR spectroscopy	Hippuric acid, 1,3-Dihydroxyphenyl-2-O-sulphate and 4-O-methylgallic acids	[64]
	Urine	HPLC-ESI-MS-MS	4-O-methylgallic acids	[58]

a Negative chemical ionization Gas chromatography–mass spectrometry

b accurate mass fragmentation

c Mass-guided SPE-trapping of selected compounds for nuclear magnetic resonance spectroscopy measurements

TMAO: Trimethylamine-N-oxide; PhIP: 2-Amino-1-methyl-6-phenylimidazo[4,5-b]pyridine; SMCSO: S-methyl-L-cysteine sulfoxide

### Biomarkers of fish intake

Many metabolomic studies have reported high levels of trimethylamine-*N*-oxide (TMAO) in urine samples following fish consumption 24 h prior to sample collection [36, 55, 65]. Lloyd and colleagues specifically searched for biomarkers of salmon in a study where subjects (*n*=24) consumed a breakfast with either one of four test foods, salmon being one of the test foods, 6 times over an 8 month period. Postprandial urine samples were collected at 3 different time points (1.5-, 3-, and 4.5-h) and analysed by flow infusion electrospray-ionisation mass spectrometry, followed by supervised data analysis in order to identify signals resulting from consumption of each test food. A combination of TMAO and 1-methylhistidine were found to be associated with salmon consumption with higher levels found after consuming the fish when compared to the standard breakfast [36].

### Biomarkers of meat intake

Meat intake is an important contributor to dietary protein in omnivorous populations and therefore has a potential impact on a range of nutritional and health outcomes [66]. As a result numerous studies, both metabolomics and non-metabolomics based, throughout the years have proposed the following metabolites as biomarkers of meat intake; creatinine, creatine, carnitine, carnosine, taurine, 1-methylhistidine and 3-methylhistidine and TMAO. A fully dietary-controlled study was analyzed by Stella and colleagues using ^1^H NMR spectroscopy in combination with multivariate statistical analysis to characterize the effects of three diets; ‘vegetarian’, ‘low meat’ and ‘high meat’ [41]. Twelve healthy male participants (24-74 years) consumed each of these diets in a randomized order for continuous 15-day periods with an intervening wash out period between each diet of 7 days duration. Three consecutive urine samples were collected from days 10-12 during each intervention period. The following metabolites were found to be increased in the high meat consumption period; creatine, carnitine, acetyl-carnithine and TMAO. Creatine is known to be influenced by a number of factors such as muscle mass hence its reliability as a biomarker needs to be further investigated. With respect to carnitine, the dietary matrix is known to have an influence on excretion so its use as a quantitative biomarker may be limited.

3-methylhistidine and 1-methylhistidine have also been proposed as biomarkers of dietary intake [42, 47, 67]. A recent study investigated both of these metabolites in conjunction with taurine and creatinine as biomarkers of meat intake [42]. This targeted analysis of urine samples following consumption of increasing amounts of red meat indicated that both 3-methylhistidine and 1-methylhistidine are good markers of red meat intake but also highlighted that 1-methylhistidine may be more useful as its excretion is independent of muscle mass and catabolism.

Previous metabolomic studies have shown TMAO to be elevated after consumption of high-meat diets [24, 41], although it has also been reported to be found in higher levels after fish consumption [36], indicating that TMAO may be used as a dietary biomarker of protein as opposed to a specific food i.e. meat/fish. A recent study assessing the effect of high or low protein diets found that the TMAO signal in the NMR spectra of urine was highly correlated to daily urinary nitrogen excretion (*r*=0.89) and thereby consumed protein [68].

### Biomarkers of fruit and vegetable intake

In recent years two groups have independently identified proline betaine as a marker of citrus fruit consumption [17, 59]. Heinzmann performed an acute intervention involving 8 volunteers where they consumed a standardised breakfast, lunch and dinner meal from day 0 until lunch on day 3. In addition to the standard dinner a supplementary mixed-fruit meal (apple, orange, grapefruit and grapes) was introduced on the evening of day 2. Urine was collected 4 times/day from the morning of day 1 until the evening of day 3. ^1^H NMR and PLS-DA analysis identified the urinary excretion of proline betaine as a biomarker of citrus fruit intake. Following on from this the authors quantified the relative concentrations of proline betaine in citrus products and evaluated the urinary excretion profile after orange juice consumption. Finally, validation was carried out on the biomarker proline betaine by using urinary NMR spectra from participants of the INTERMAP UK cohort [69]. A receiver operating characteristic (ROC) curve resulted with an AUC of 92.3% with a sensitivity and specificity of 90.6% and 86.3% respectively.

In the study performed by Lloyd and colleagues proline betaine was identified as a biomarker of citrus intake using an acute breakfast challenge. Acute exposure of volunteers to orange juice resulted in the appearance of proline betaine and a number of biotransformed products in postprandial urine samples. In addition, a process of validation showed sensitivities of 80.8-92.2% and specificities of 74.2-94.1% for elevated levels of proline betaine in those volunteers who reported a high consumption [59].

Applications of a metabolomics strategy for the identification of biomarkers of cruciferous vegetable consumption has recently identified S-methyl-L-cysteine sulphoxide (SMCSO) and metabolic derivatives as putative biomarkers [37]. Twenty healthy male subjects (*n*=20) were recruited to a three period dietary intervention study with each period lasting 14 days. For phases I and III a low cruciferous vegetable intake was consumed, whereas phase II consisted of a high cruciferous vegetable intake. On day 13 of each phase, following an overnight fast, a time zero spot urine sample was obtained from each participant in the study. Participants were then maintained on a standardised diet and urine sample collections were obtained for the periods 0-10, 10-24 and 24-48 h. Analysis of the NMR spectra showed clear differentiation between the high and low cruciferous vegetable consumption and was attributed to SMCSO and metabolites derived from it.

Other candidate biomarkers for fruit and vegetable intake include antioxidant vitamins such as vitamin C [70, 71] and flavanoids [58], including quercetin [72]. Mennen *et al*. examined associations between dietary intakes and the concentrations of selected urinary polyphenols and metabolites in free living subjects [58]. In this study 13 polyphenols and metabolites were measured in urine samples using HPLC-ESI-MS-MS along with two day food diaries which estimated habitual intake. In spot urine samples, significant correlations were reported for different fruits and beverages and several polyphenol compounds, for example apple consumption was positively correlated to phloretin, grapefruit consumption to naringenin, orange to hesperetin, citrus fruit consumption to both naringenin and hesperetin, with *r* coefficients ranging from 0.31 to 0.57 (P < 0.05).

### Biomarkers of tea consumption

Tea is a widely consumed beverage and is a major dietary source of polyphenolic compounds, including phenolic acids and flavanoids. Several potential biomarkers of exposure to tea derived polyphenols have been identified [73–75]. These include specific *O*-methylated polyphenols derived from in *vivo* polyphenol metabolism, such as 4-O-methylgallic acid (4OMGA) [73, 76, 77]. One such study explored the relationship of 24 h urinary excretion of 4OMGA with usual (*n*=111) and current (*n*=344) tea intake in human subjects using a GC-MS approach [75]. The authors found that urinary excretion was significantly related to both usual tea intake (*r* 0.50, P < 0.001) and current tea intake (*r* 0.57, P < 0.001) and that a cut-off concentration for 4OMGA excretion of 25 µg/mmol creatinine had 82% sensitivity and 81% specificity for prediction of tea drinking status.

### (ii) Dietary patterns and metabolomic profiles of habitual diet to identify nutritypes and biomarkers

The studies described above have efficiently identified biomarkers of certain foods. In recent years there has been an interest in dietary patterns and their use as a method of studying relationships between diet and disease.

Work in our laboratory applied dietary pattern analysis to 125 subjects for which dietary data was recorded using 3 day food diaries [43]. The identification of dietary clusters was performed using *k*-means clustering and resulted in three cluster groups which were associated with unique food intakes and differed in aspects of their nutrient intake profiles. Assessment of the metabolomic profiles revealed that the cluster groups were reflected in the urinary metabolomic profiles. Further analysis using PLS-DA identified metabolites associated with the different dietary patterns. Cluster 3 was defined by high intakes of meat products, white bread, butter and preserves and had significantly higher levels of *O*-acetylcarnitine. The novelty of this work lies in the fact that identification of nutritypes (i.e. metabolic profiles that reflect dietary intake) has the potential to aid dietary assessment by unobjectively classifying people into certain dietary patterns.

Peré-Trepat and colleagues also developed a strategy for assessing links between dietary data (FFQ) and metabolomic profiles [78]. In this work dietary patterns were defined by PCA and then re-coded and regressed against NMR metabolic profiles to obtain loadings and identify metabolites associated with dietary patterns. While this study was a method development study it successfully linked dietary patterns with certain metabolites and further supports the concept of nutritypes.

Using the KORA (Cooperative Health Research in the Region of Augsburg) study population, Altmaier *at al*. identified seven dietary patterns [79]. Metabolomic analysis was performed on plasma samples using electrospray ionization (ESI) tandem mass spectrometry (MS-MS). Statistical analysis revealed that certain dietary patterns were highly associated with serum metabolite concentrations.

Overall, these studies provide good evidence for the potential of metabolomics to be used to define a profile of markers that are reflective of a habitual dietary pattern. Further studies will be necessary to develop this concept further and establish its robustness across different populations.

## Summary and Outlook

Although biomarkers cannot replace traditional dietary assessment methods, the use of metabolomics in identifying novel and robust biomarkers of dietary exposure and intake can enhance and validate such methods. Additionally the use of metabolomics in characterising habitual dietary exposure and the identification of nutritypes is an interesting and emerging field with potential applications in nutrition epidemiology.

For metabolomics to reach its full potential in this field a number of challenges need to be addressed. Examples of these challenges include a requirement for technology advancement to enhance our metabolite coverage and advancement in the identification of unknown metabolites to allow novel biomarker discovery. Finally cooperation across disciplines is required to ensure optimal usage of dietary biomarkers.
